# Draft Genome Sequence and Gene Annotation of *Stemphylium lycopersici* Strain CIDEFI-216

**DOI:** 10.1128/genomeA.01069-15

**Published:** 2015-09-24

**Authors:** Mario E. E. Franco, Silvina López, Rocio Medina, Mario C. N. Saparrat, Pedro Balatti

**Affiliations:** a Centro de Investigaciones de Fitopatología (CIDEFI-CICBA), Facultad de Ciencias Agrarias y Forestales, Universidad Nacional de la Plata, La Plata, Buenos Aires, Argentina; b Instituto de Fisiología Vegetal (INFIVE-CCT La Plata-CONICET), Facultad de Ciencias Agrarias y Forestales, Facultad de Ciencias Naturales y Museo, Universidad Nacional de La Plata, La Plata, Buenos Aires, Argentina; c Centro de Investigación y Desarrollo en Fermentaciones Industriales (CINDEFI-CCT La Plata-CONICET), Facultad de Ciencias Exactas, Universidad Ncional de La Plata, La Plata, Buenos Aires, Argentina; d Instituto Carlos Spegazzini, Facultad de Ciencias Naturales y Museo, Universidad Nacional de La Plata, La Plata, Buenos Aires, Argentina; e Cátedra de Microbiología Agrícola, Facultad de Ciencias Agrarias y Forestales, Universidad Nacional de La Plata, La Plata, Buenos Aires, Argentina

## Abstract

*Stemphylium lycopersici* is a plant-pathogenic fungus that is widely distributed throughout the world. In tomatoes, it is one of the etiological agents of gray leaf spot disease. Here, we report the first draft genome sequence of *S. lycopersici*, including its gene structure and functional annotation.

## GENOME ANNOUNCEMENT

Dematiaceous fungi belonging to the genus *Stemphylium* Wallr., a taxonomical name established in 1833, produce single-cell conidia (often dictyoconidia) in a percurrent conidiogenous cell. This genus is the anamorphic stage of the teleomorph *Pleospora* (*Pleosporaceae*, *Pleosporales*, *Dothideomycetes*, ascomycetes) (1, 2). Currently, the number of described *Stemphylium* species varies from 30 to 150 (2, 3). Fungi within this genus are widely distributed and have a broad plant host range, establishing a pathogenic, saprotrophic, or entophytic relationship with their hosts (4–7). Pathogenic forms cause severe yield reduction and economic losses in horticultural and fruit tree crops (2, 8). Since *Stemphylium lycopersici* was first described on tomatoes (*Solanum lycopersicum* L.) in 1931 (9, 10), it has been considered the causal agent of leaf spot in >30 host genera worldwide (8, 11). Here, we report the first whole-genome shotgun sequence of the plant-pathogenic fungus *S. lycopersici* strain CIDEFI-216.

*S. lycopersici* strain CIDEFI-216 was isolated from a tomato plant affected by gray leaf spot disease collected at the tomato-growing area of Bella Vista, Corrientes, Argentina, in 2010. Total genomic DNA was isolated from a monosporic culture using the DNeasy plant minikit (Qiagen). The quality of the DNA was assessed by gel electrophoresis, and the quality and quantity were determined by the NanoDrop 2000 UV-Vis spectrophotometer (Thermo Scientific). Libraries were prepared with the TruSeq Nano DNA library preparation kit, 2 × 100-bp paired-end sequencing was performed using an Illumina HiSeq 2000 sequencing system, and reads were assembled with the SOAP*denovo*2 assembler software (12) at Macrogen Co. (Seoul, South Korea). *Ab initio* gene prediction was performed using Fgenesh 2.6 (13), trained on an algorithm optimized for *Alternaria brassicicola*. tRNAs and rRNAs were predicted using the tRNAscan-SE (14) and hmmsearch 3.0 (15–18) tools from the WebMGA server (19). A homology-based automatic annotation of the predicted open reading frames (ORFs) was carried out using Blast2GO Basic (20) (BLASTp against the NCBI nonredundant protein database with an e-value cutoff of 1E-6 and a maximum of 20 BLAST hits) and InterProScan (21); gene names, Gene Ontology (GO) terms, Enzyme Commission (EC) numbers, and InterPro codes were assigned.

The shotgun sequencing yielded 3.14 Gbp of raw data, which represents 60-fold genome coverage. The genome was assembled into 414 scaffolds (>1,000 bp; *N*_50_, 498,048 bp), with a total length of 35.18 Mbp and an overall G+C content of 50.8%. A total of 8,999 protein-coding genes were predicted; among them, 6,885 were assigned with 26,524 GO terms (42.8% GO function, 40.7% GO process, and 16.5% GO component), 1,526 with 1,723 EC numbers, and 8,169 with 99,069 InterPro codes from all databases that make up the InterPro Consortium. Additionally, 94 tRNAs and 16 rRNAs were found.

The *S. lycopersici* draft genome sequence generated in this study represents a new source of knowledge that will be useful to further understand the molecular mechanisms of its pathogenesis and other lifestyles. It will also be valuable in comparative genomic studies to shed light into the taxonomy of this fungus.

### Nucleotide sequence accession numbers.

This whole-genome shotgun project has been deposited at DDBJ/EMBL/GenBank under the accession no. LGLR00000000. The version described in this paper is version LGLR01000000.
